# hCG and Its Disruption by Environmental Contaminants during Human Pregnancy

**DOI:** 10.3390/ijms19030914

**Published:** 2018-03-20

**Authors:** Luana Paulesu, Ch.V. Rao, Francesca Ietta, Adalgisa Pietropolli, Carlo Ticconi

**Affiliations:** 1 Department of Life Sciences, University of Siena, 53100 Siena, Italy; francesca.ietta@unisi.it; 2 Department of Cellular Biology and Pharmacology, Herbert Wertheim College of Medicine, Florida International University, Miami, FL 33199, USA; crao@fiu.edu; 3 Department of Biomedicine and Prevention, Section of Gynecology and Obstetrics, Tor Vergata University, 00173 Rome, Italy; pietropolli@med.uniroma2.it (A.P.); ticconi@uniroma2.it (C.T.)

**Keywords:** endocrine disrupting chemicals, Bisphenol A, *para*-Nonylphenol, human placenta, human trophoblast

## Abstract

Human chorionic gonadotropin (hCG) is a hormone of considerable importance in the establishment, promotion and maintenance of human pregnancy. It has been clearly demonstrated that hCG exerts multiple endocrine, paracrine and autocrine actions on a variety of gestational and non-gestational cells and tissues. These actions are directed to promote trophoblast invasiveness and differentiation, placental growth, angiogenesis in uterine vasculature, hormone production, modulation of the immune system at the maternal-fetal interface, inhibition of myometrial contractility as well as fetal growth and differentiation. In recent years, considerable interest has been raised towards the biological effects of environmental contaminants, particularly endocrine disrupting chemicals (EDCs). Emerging evidence suggests that prenatal exposure to selected EDCs can have a deleterious impact on the fetus and long-lasting consequences also in adult life. The results of the in vitro effects of commonly found EDCs, particularly Bisphenol A (BPA) and *para*-Nonylphenol (*p*-NP), indicate that these substances can alter hCG production and through this action could exert their fetal damage, suggesting that hCG could represent and become a potentially useful clinical biomarker of an inappropriate prenatal exposure to these substances.

## 1. Introduction

Human Chorionic Gonadotropin (hCG) is a molecule of considerable biological importance, being implicated, nearly in all the major reproductive and developmental processes in humans. The knowledge on the biological and evolutionary significance, as well as the functional importance of hCG, has been enormously expanded in the last few decades. Consequently, many new concepts on the role of hCG have been added to those traditionally established in the fields of reproductive and non-reproductive biology. Moreover, these new concepts have led to many new potential therapies with hCG. The therapies are for preterm labor, painful bladder syndrome/interstitial cystitis, overactive bladder, chronic pain, breast cancer, HIV/AIDS, rheumatoid arthritis, Sjogren’s syndrome and few other autoimmune diseases and tubal infections with *Neisseria Gonorrhoeae* [1]. Some of these therapies have been validated in women (preterm labor, painful bladder syndrome/interstitial cystitis, chronic pain) and the others in animal models [1]. All of them are waiting for validation by randomized placebo controlled clinical trials.

One of the modern frontiers in hCG study is to investigate the effects of hCG disruption in human pregnancy, which can be a consequence of, or lead to, very different pathologic conditions. Abnormalities in the production and the circulating levels throughout specific periods of gestation and in the relative variations of the several glycoforms of hCG have been associated with a large array of pregnancy complications, such as miscarriages [2], fetal chromosomal anomalies [3], preeclampsia [4,5], disturbances in fetal growth and development [6], gestational trophoblast diseases [7], etc.

In the last two decades, interest has been growing on the potential role of several environmental contaminants on the secretion of hCG as well as on the fetal well-being and the pregnancy outcome.

The objective of this article is to review the major currently recognized roles of hCG in pregnancy and to examine the effects of selected environmental contaminants that have been shown to have the capacity to impact the regulation of hCG secretion.

## 2. The hCG Molecules

hCG is an extremely complex molecule. Based on the structural and/or functional similarities, hCG can be included in three families. First is the glycoprotein hormone family, which consists of follicle stimulating hormone (FSH), luteinizing hormone (LH) and thyroid stimulating hormone (TSH). Second is the cystine-knot growth factor family, which includes nerve growth factor, platelet derived growth factor, fibroblast growth factor beta and transforming growth factor-beta. The third is the therapeutic glycoproteins family, which contains prominent members like erythropoietin, interferons, monoclonal antibodies and tissue plasminogen activator [8]. Like these therapeutic glycoproteins, hCG has an unrealized potential in saving the countless number of human lives from some of the most dreadful diseases [1,8].

hCG has endocrine, paracrine and autocrine actions [9]. It is a heterodimeric glycoprotein composed by two subunits—α and β—and is considered a more active agonist of LH receptors [10]. The α subunit is common with TSH, FSH and LH and is encoded by a single gene (CGA) [9], whereas the β subunit of each of the above hormones is encoded by different genes. In case of the hCG beta subunit, a cluster of six non-allelic genes: *CGB1*, *CGB2*, *CGB3*, *CGB5*, *CGB7* and *CGB8* are involved [9]. hCG β subunit is similar to that of LH, it shares 82% homology [11], and binds to the same G-protein coupled LH/hCG receptors [12]. However, there is convincing evidence that the downstream intracellular effects of hCG on relevant pathways such as the adenylate cyclase/protein kinase A, the phospholipase C/inositol phosphate, the AKT and the ERK 1/2 are different from those of LH after receptor binding [13,14,15]. Therefore, emerging evidence shows that important differences exist between LH and hCG. LH is present in all vertebrates, whereas hCG is found only in primates, which strongly suggests that LH is a strongly conserved molecule during evolution, whereas hCG is a relatively recently evolved molecule from LH [13].

hCG is a highly glycosylated molecule. In fact, 30% of its molecular weight is represented by carbohydrate moieties [16,17]. There are two *N*-glycosylation sites in the α-subunit, whereas the β-subunit of hCG contains two *N*-glycosylation and four *O*-glycosylation sites [9,16,17]. Currently, at least 5 variant glycoforms of hCG have been detected: hCG, sulphated hCG (S-hCG), hyperglycosylated hCG (H-hCG), hCG free β-subunit (free β-hCG) and hyperglycosylated hCG free β-subunit (H-free β-hCG). hCG variants can be generated by the degree, type as well as the location of carbohydrate moieties on the protein back bone of the molecule. The glycosylation is responsible for the hCG glycoforms circulating half-life (much longer for hCG than for LH) and biological activity [14,15,16,17]. It has been reported that pregnancy can be detected by an abundance as well as by glycoforms of hCG [18].

## 3. Major Roles of hCG during Pregnancy

There is no doubt that hCG is a fundamental molecule for human pregnancy and of its actions are directed towards pregnancy maintenance [19]. In fact, it became clear that hCG can act as an endocrine as well as a paracrine/autocrine molecule. The classic actions of hCG—i.e., ovulation induction (in addition to LH), maintenance of the corpus luteum and stimulation of its progesterone production during the first 9 weeks of pregnancy—are exerted through its binding to the LH/hCG receptors [13,18,20]. Additional recently discovered hormonal actions of hCG are inhibition of myometrial contractility [20,21], stimulation of angiogenesis in uterine vasculature [22], promotion of umbilical circulation and placental growth [23] as well as many different immunomodulatory effects [17,24,25,26,27,28,29]. They allow for an establishment and prolongation of maternal tolerance towards the fetal semiallograft [17,30]. Moreover, it has been shown that several fetal organs like kidney, liver, lung, spleen, bowel contain LH/HCG receptors [31]. These organs in adults are devoid of them, suggesting that hCG could play a role in their growth and development. There is a consensus that the endocrine actions of hCG are exerted by receptor binding followed by an activation of several intracellular signalling pathways, such as adenylate cyclase/cAMP/PKA and the ERK1/2 MAPK pathways [15,32].

Together with these endocrine actions of hCG, several important autocrine and/or paracrine effects of hCG have now been unravelled: stimulation of cytotrophoblasts differentiation into syncytiotrophoblasts [33], increased progesterone secretion by syncytiotrophoblasts [34,35], potent in vitro angiogenic effect [36], etc.

## 4. Environmental Contaminants in Human Pregnancy: Implications for the Fetus

Much attention has been paid in the last several decades on the possible impact of environmental contaminants, particularly endocrine disrupting chemicals (EDCs), on human pregnancy [37]. Maternal contamination with these substances has been linked to several adverse consequences for pregnancy and fetal health [38,39]. An important class of EDCs shares many similarities with the endogenous estrogens and can interfere with the role of these important hormones in reproductive processes. Like natural estrogens, these compounds act in fact on estrogen-responsive organs by binding to ERα and ERβ and regulating target gene expression [40]. They can also activate non genomic pathways and induce more rapid responses through activation of multiple kinases [41]. Estrogens are fundamental hormones in pregnancy, therefore any interference with their action might be harmful for pregnancy and fetal health.

The estrogen-like EDCs include a broad range of man-made chemicals used in industrial lubricants, solvents, several pesticides, biocides, plasticizers, surfactants, pharmaceutical agents. Some naturally occurring EDCs can also be found in plants or fungi.

Given the widespread distribution of these chemicals in the environment and their presence in commonly used materials, it appears almost inevitable for humans to avoid their contamination. The high human exposure is demonstrated by the EDCs levels found in tissues and body fluids of most inhabitants in agricultural and non-industrialized areas [42,43]. Most of these chemicals can also pass through the placenta and reach the fetus, as revealed by the concentrations in amniotic fluid, cord blood and placental and fetal tissues [44,45].

Prenatal life is a critical and vulnerable period during which the environmental insults may lead to permanent changes in cells, tissues and hence in the whole organs [46]. According to the “Barker hypothesis”, also known as “Fetal origins of adult disease hypothesis”, any adverse influence early in the development and particularly in the intrauterine life can result in a variety of disorders that may become manifest later in life [47]. This can actually occur in the case of EDCs.

Among the health disorders that can be potentially attributed to EDCs are type 2 diabetes, several metabolic and neurologic disorders as well as reproductive defects and increased cancer risk [48,49,50,51]. It is therefore essential to fully understand how these chemicals act during the intrauterine life and to prevent complications in new-borns as well as in their adult life. In this context, emerging experimental evidence suggests that interference with hCG production by the placenta could be a way by which selected EDCs can exert their effects on the fetus. This issue will be discussed in detail in the following section.

## 5. In Vitro Effects of Selected EDCs on Human Placenta

The placenta, which is interposed between fetal organs and the maternal circulation, transfers substances and gases. In addition, placenta is an active organ involved in the metabolism and synthesis of hormones fundamental to pregnancy establishment and development. The placenta also acts as an immune barrier protecting the fetus against maternal rejection. However, the placenta is not adapted to act as a barrier to the many man-made chemicals commonly present in the environment [52]. Therefore, many environmental contaminants elude placental barrier and potentially harm the fetus. In addition, the placenta is a highly sensitive organ to environmental contaminants with estrogenic activity as it expresses ERα and ERβ [53]. It is conceivable that while passing through placenta, EDCs can act on it as well. Given the key role of placenta in maintenance of pregnancy and assuring the fetal growth and development, any alterations in placental functions, caused by exposure to potentially harmful EDCs, may lead to dangerous consequences to the health of pregnancy and fetus.

Many efforts have been made to test the effects of EDCs in human placenta. In vivo administration cannot be used in human pregnancy, for obvious ethical reasons. On the other hand, animals are not completely suitable for studies on the placenta because this organ varies from one species to another. Therefore, in vitro models of human placenta have become key to evaluate the potential damaging effects of EDCs. These are mainly represented by established trophoblast cell lines and primary cultures of isolated trophoblasts or placental explants [54].

Although there are many reports in the literature on the in vitro action of different EDCs in human placenta, some controversies remained particularly regarding the timing, dose and duration of exposure. We discuss here only experimental studies involving low non-toxic doses of EDCs with emphasis on Bisphenol A (BPA) and *para*-Nonylphenol (*p*-NP), two EDCs that attracted the attention by many researchers and including our laboratory during the last ten years. However, when appropriate, effects of other chemicals on human placenta will be also discussed.

BPA is one of the highest volume EDC produced worldwide and a component of many everyday products including plastic bottles, detergents and food packaging. Although its use in items for children aged 0–3 was banned (European Commission 2011; U.S. Food and Drug Administration (FDA) 2012), BPA is still used in many other products so that its presence is largely documented in the environment and as well as in the general populations [55].

In collaboration with a Danish group, the research group of the University of Siena, co-author in this review, showed that BPA is transferred through placenta [56]. In vitro studies on BeWo cells and chorionic villous explants from human placenta have demonstrated that low BPA concentrations, that do not affect the cell viability, have adversely affected key functional processes of placental development. In particular, BPA was found to increase the secretion of β-hCG and cell apoptosis, two markers of syncytialization/differentiation of the epithelial layer of chorionic villi, which is in direct contact with maternal blood [56]. By using the HTR-8/SVneo, an invasive cell type representative of the extravillous trophoblast, it was demonstrated that very low BPA concentrations reduced the cell migration and invasion, without affecting the cell proliferation [57]. A study on primary trophoblasts isolated from term pregnancy placentas showed that BPA also induced 11b-HSD2 activity, protein and mRNA, aromatase, glucose transporter-1, CRH and hCG mRNA levels [58].

Several reports compared the effect of different EDCs under similar experimental conditions. In our studies, the effect of BPA was compared with that of *p*-NP, a potentially hazardous chemical known to have estrogenic activity since 1991 [59]. *p*-NP is mainly used as plasticizer and surfactant in the manufacturing industry and present in detergents, paints, pesticides, personal care products, and food and drink packaging. The in vitro studies showed that similar to BPA, *p*-NP could increase β-hCG secretion, cell apoptosis and reduce trophoblasts migration and invasion [57]. The studies on cell migration/invasion also revealed that *p*-NP was more potent than BPA, because it is active at lower concentrations and after a shorter duration of exposure [57]. *p*-NP exerted also a statistically significant effect in reducing trophoblast/endothelial cells interaction, while BPA did not.

A key role of human placenta is to protect the fetus against potentially harmful substances. This is accomplished by transporter proteins in the epithelium of chorionic villi, which move away chemicals from the fetal circulation [60]. Insufficiency of these transporters can have effects on the clearance of molecules, impacting fetal growth and development [60]. In a collaborative study with a research group in Kuopio, Finland, the research group of Siena showed that exposure of placental explants to BPA and *p*-NP down-regulated expression of ABCG2, a key efflux ABC (ATP-binding cassette) transporter for xenobiotics [61]. Activity of both chemicals was at 1 nM while higher concentration (100 mM) did not have a significant effect. Importantly, ABCG2 showed changes dependent on the gestational age with a higher expression in tissues at first trimester than at term pregnancy and susceptibility to chemicals only in tissues at term [61].

It is important to emphasize that the effects of EDCs in human trophoblasts are dose-dependent with low doses being more effective than high doses [62]: This fact causes very great concern because the efficacious low doses correspond to the levels detected in the human population. With regard to *p*-NP, the highest dose of 1 nM corresponds to 0.220 ng/mL, a similar value recovered in human blood samples [63]. Concerning BPA, 1 nM corresponds to 0.228 ng/mL with cconcentrations of BPA ranged from 0.3 to 18.9 ng/mL in maternal plasma [44,45].

## 6. EDCs and hCG

The particular effects of EDCs at low, non-toxic doses, have been better demonstrated by measuring hCG secretion in cultures exposed to different compounds. Among the other endpoints of EDCs in human placenta, hCG has the advantage of being a measurable parameter, thus allowing to detect even subtle changes caused by different EDC concentrations. For this purpose, studies had been performed with different chemicals in a large range of concentrations, not affecting cell viability. These chemicals included a pesticide, (Atrazine), a non-steroidal estrogenic compound used in the past as a therapeutic tool for reproductive disorders (Diethylstilbestrol), a phytoestrogen (Resveratrol) as well as BPA and *p*-NP [56,64,65]. The studies showed that all chemicals tested altered β-hCG secretion in a dose dependent U-shaped curve with Atrazine, BPA and *p*-NP and an inverted U-shaped curve with Diethylstilbestrol and Resveratrol.

This nonlinear behavior of EDCs revealed that chemicals were stimulating or inhibiting β-hCG secretion, depending on their concentration. As reported in Table 1, concentrations as low as 0.1 pM could inhibit β-hCG secretion in the case of Diethylstilbestrol and Resveratrol, while *p*-NP had a stimulating effect. For a more complete information, this table includes data on an insecticide (Chlorpyrifos), two isomers of 1,1,1,-trichloro-2,2-bis(*p*-chlorophenil)ethane) (*p*,*p*′-DDT and *o*,*p*′-DDT) and their metabolites 1,1,-dichloro-2,2-bis(*p*-chlorophenyl)ethylene (*p*,*p*′-DDE and *o*,*p*′-DDE), although these substances have been tested only at a narrow range of concentrations [66,67]. These data also show differences in hCG secretion depending on the duration of exposure to these chemical agents. 

## 7. hCG as a Potential Biomarker of EDCs Action in Human Pregnancy

Altogether the data on EDCs in human pregnancy show that maternal exposure to lower concentrations corresponding to the levels detected in the human populations may harm placental development and hormone (hCG) secretion in a concentration- and time-dependent manner. Although it is difficult to translate the data obtained in vitro into the pathophysiological conditions, placental dysfunction could be a contributory cause of the adverse pregnancy outcomes reported with maternal contamination by EDCs. These studies show association of EDCs levels in maternal blood and/or urine with increasing risk of miscarriages [68,69], preterm birth [70,71], reduced birth weight [72] and increased risk of preeclampsia [73,74]. Despite the well demonstrated impact of EDCs on human placenta, the current available information on placental biomarkers—particularly on measurable parameters during pregnancy—in the above conditions is scant. Ferguson et al., 2015 [75] investigated the possible association between selected plasma biomarkers of angiogenesis—namely fms-like tyrosine kinase-1 (sFlt-1) and Placental growth factor (PlGF)—and urinary biomarkers of exposure to phthalate and BPA. The authors found a positive association between BPA levels and an increase of plasma sFlt-1 as well as an increase in the ratio of sFlt-1 to PlGF [75]. These findings are suggestive of an altered placentation and trophoblast function. In other studies, the same group showed an association of EDCs exposure during pregnancy with plasma biomarkers of inflammation and/or oxidative stress [76]. These observed effects might be the result of activity of EDCs in trophoblast as these substances, BPA and NP, have been shown to induce generation of reactive oxygen species (ROS) in JEG-3 cells [77].

Nevertheless, we are not aware of any information on hCG blood level in association with maternal contamination by EDCs, in pregnancies complicated by disorders. The only exception is the use of serum β-hCG level <6 mIU/mL for identification of implantation failure, in women undergoing in vitro fertilization [78]. Low hCG level is indeed a marker of early miscarriages [79,80]. By contrast, preeclampsia, a severe disease occurring in late gestation is linked to high levels of hCG [81]. Serum β-hCG measurement is not only diagnostic but also has a good predictive value for pregnancy outcome.

Considering the importance of hCG levels, and on the basis of the in vitro data showing disruption of hCG secretion by exposure to EDCs as well as the association of maternal contamination by EDCs with pregnancy complications, we suggest altered serum hCG concentration as a link between maternal exposure to EDCs and adverse pregnancy outcomes.

**Figure 1:**
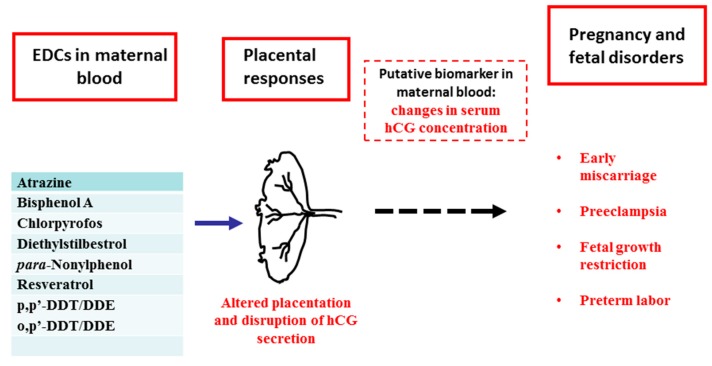
Potential mechanisms by which EDCs could alter placental development and hCG production and affect pregnancy outcome: changes in maternal serum hCG concentration could act as biomarkers of EDCs action.

An hypothetical scenario is graphically represented in Figure 1: (a) The placenta is a sensitive target of environmental EDCs in maternal blood; (b) in response to these chemicals, the placenta undergoes changes in its development and hormone (hCG) secretion; (c) placental dysfunction and/or any resulting change in maternal blood may lead to various pregnancy and fetal disorders. 

The in vitro data discussed here allows us to propose that changes in hCG maternal serum concentration might be a putative biomarker of EDCs actions during pregnancy.

Of course, future clinical-experimental studies should be addressed to examine the possible association between EDCs levels in maternal blood and serum hCG concentration, at different times of pregnancy and in relation to pregnancy and/or fetal disorders.

## 8. Concluding Remarks

There is a growing evidence on the potential dangerous effects of prenatal exposure to environmental contaminants, particularly EDCs, on the pregnancy outcome and the fetal development. These substances can diffuse into placenta and end up in fetus. Nearly everybody comes in contact with them in the everyday life. Research in this field is at the beginning, despite a large number of studies have already been published. In fact, several environmental contaminants have been demonstrated or strongly suspected to have short-term adverse effects on the fetus, the neonate and possibly long-term effects also in the adult life. However, the debate is still continuing [82,83], not only on many specific substances but also on the mechanisms and pathways involved in the genesis of the damage [84,85]. The in vitro studies carried out on the effects of selected EDCs on human trophoblasts indicate that these substances can exert significant effects on the production and release of hCG, which is a major molecule promoting pregnancy. Therefore, it is not unreasonable to hypothesize that maternal contamination with EDCs could disrupt placental endocrine activity which, in turn, could lead to changes in hCG concentration in maternal blood.

Further studies are needed to fully confirm this hypothesis which, if validated, could lead to consider hCG could serve not only as an expression of fetal well-being, but also serve as a useful clinical biomarker in determining the degree of prenatal exposure to EDCs.

## Figures and Tables

**Table 1:** Disruption of hCG (human chorionic gonadotropin) secretion by human placenta/trophoblast cells exposed to various EDCs (endocrine disrupting chemicals) at different concentrations and times.

	hCG Secretion	Reference
**Atrazine**	Decrease in BeWo cells at 10 pM–1 nM	[64]
	Increase in BeWo cells at of 0.1–1 mM	[64]
**BPA**	Decrease in BeWo cells at 30 μM	[56]
	Increase in primary trophoblast cells from human placenta term at 0.44, 1.1, 2.2, 4.4 or 8.8 µM	[58]
	Increase in placental explants from first trimester placenta at 1 or 0.5 nM	[65]
**Chlorpyrifos**	Increase in primary trophoblast cells from human placenta at term at 50 or 100 μM	[66]
**DES**	Decrease in BeWo cells at 0.1 pM, 10 nM or 0.1 µM	[64]
***p*-NP**	Increase in BeWo cells at 0.1 pM	[64]
	Decrease in BeWo cells at 10 pM–1 nM	[64]
	Increase in placental explants from first trimester placenta at 1 nM	[53]
**Resveratrol**	Decrease in BeWo cells at 0.1–1 pM	[64]
***p*,*p*′-DDT/DDE**	Decrease in JEG3 cells at 1, 10, 100 ng/mL, 1 µg/mL after 24 h	[67]
***o*,*p*′-DDT/DDE**	Increase in JEG3 cells at 1, 10, 100 ng/mL, 1 µg/mL after 72 h	[67]
